# Miniaturized Wideband Loop Antenna Using a Multiple Half-Circular-Ring-Based Loop Structure and Horizontal Slits for Terrestrial DTV and UHD TV Applications

**DOI:** 10.3390/s21092916

**Published:** 2021-04-21

**Authors:** Junho Yeo, Jong-Ig Lee

**Affiliations:** 1 School of ICT Convergence, Daegu University, 201 Daegudae-ro, Gyeongsan, Gyeongbuk 38453, Korea; 2 Department of Applied Electronics Engineering, Dongseo University, San69-1, Jurye-2dong, Sasang-gu, Busan 47011, Korea; leeji@gdsu.dongseo.ac.kr

**Keywords:** miniaturized, wideband loop antenna, digital television, half-circular rings, horizontal slits

## Abstract

A miniaturized wideband loop antenna for terrestrial digital television (DTV) and ultra-high definition (UHD) TV applications is proposed. The original wideband loop antenna consists of a square loop, two circular sectors to connect the loop with central feed points, and a 75 ohm coplanar waveguide (CPW) feed line inserted in the lower circular sector. The straight side of the square loop is replaced with a multiple half-circular-ring-based loop structure. Horizontal slits are appended to the two circular sectors in order to further reduce the antenna size. A tapered CPW feed line is also employed in order to improve impedance matching. The experiment results show that the proposed miniaturized loop antenna operates in the 460.7–806.2 MHz frequency band for a voltage standing wave ratio less than two, which fully covers the DTV and UHD TV bands (470–771 MHz). The proposed miniaturized wideband loop antenna has a length reduction of 21.43%, compared to the original loop antenna.

## 1. Introduction

Digital television (DTV) broadcasting has become widespread, replacing conventional analog TV broadcasting due to such advantages as a high transmission rate with high spectrum efficiency, multi-channel operation, and high picture quality. The transition from analog to digital broadcasting in Korea began in 2000 and was completed at the end of 2012 [1]. From 2013 onwards, the DTV broadcasting frequency band was reduced from 470–806 MHz to 470–698 MHz in order to reflect the demand for additional bands in mobile and disaster communications. Among them, the 698–710 MHz and 753–771 MHz bands are used for terrestrial ultra-high definition (UHD) TV [2]. Therefore, an antenna for terrestrial DTV and UHD TV reception needs to receive signals in the 470–771 MHz frequency band (48.5%) and must have a wideband frequency characteristic. The antenna should use horizontal polarization based on the ground [3] and be designed based on 75 ohms, because a broadcasting coaxial cable is used for the feed line [4].

Antenna types commonly used for indoor terrestrial DTV reception include loop, dipole, monopole, log-periodic dipole array (LPDA), and quasi-Yagi antennas [5]. The quasi-Yagi and LPDA antennas have high gain from using multiple dipoles, but it is necessary to check the receiving direction and adjust the location because of directivity [6,7,8,9]. On the other hand, the omni-directional dipole, monopole, and loop antennas can receive from all directions, but have low gain [10,11,12,13,14,15,16,17].

A broadband compact quasi-Yagi antenna (consisting of a dipole fed by a coplanar strip line, a rectangular patch-type director, and a ground reflector) was designed to cover the 450–848 MHz frequency band with moderate gain of 3.5–4.6 dBi and a high front-to-back ratio greater than 10.4 dB [6]. The size of the antenna was 240 mm × 200 mm. A 10-element LPDA antenna using Koch fractal geometries was investigated for reduction in the dipoles’ length in a standard LPDA antenna [7]. It covered the 460–1270 MHz frequency band with gain of 4.74–6.23 dBi and a 290 mm × 221 mm antenna size. A three-element Yagi-Uda antenna with a gain of 4 dBi and a six-element LPDA antenna with gain of 5 dBi operating in the DTV band were developed for telecommunication engineering education [8]. The size of the Yagi-Uda antenna was 226 mm × 206 mm, whereas that of the LPDA antenna was 283 mm × 248 mm. An LPDA DTV reception antenna with UHF mobile communications band rejection capability was proposed [9]. Ten dipole elements were used, and the lengths of the front short three dipoles and their spacing were optimized by using the trusted region framework algorithm in CST Microwave Studio in order to obtain rejection in the frequency range of 810–960 MHz. The physical dimensions of the optimized LPDA antenna were 356 mm × 303 mm. The first dipole was longer than the second dipole, and the second dipole was longer than the third dipole. The fourth dipole was longer than the third dipole.

A compact half-bow-tie-shaped dipole antenna with a modified balun was proposed for indoor DTV reception [10]. Gain greater than 0 dBi was achieved with an antenna size of 178 mm × 95 mm. A compact bent dipole antenna with two L-shaped metal stubs close to feeding point and two notched coupling strips was introduced for DTV reception with a size of 200 mm × 20 mm [11]. However, direct connection with a coaxial feed line was necessary. A planar dipole antenna with a number of notch slits with different lengths was introduced to increase the frequency bandwidth [12]. It operated at 452–897 MHz with gain of 2.1–3.9 dBi and a 250 mm × 45 mm antenna size. An unbalanced slot-printed dipole antenna with a triangular parasitic element for a DTV receiver was proposed with a size of 250 mm × 135 mm [13]. It covered 441–890 MHz with peak gain of 4.7 dBi. A planar monopole antenna with a gap sleeve was developed to operate in the 432–827 MHz frequency band with peak gain of 2.2 dBi and a 213 mm × 40 mm antenna size [14]. The outer gap sleeve was appended to the original inner sleeve of the monopole antenna to extend the impedance bandwidth. A flexible printed sleeve monopole antenna was proposed for DTV reception [15]. The antenna consisted of a meander-line folded monopole and a sleeve connected with the ground plane. It was printed on a Kapton polyimide film with a total thickness of 0.3 mm and the antenna size was 180 mm × 50 mm.

A commercial indoor DTV circular loop antenna from SPECTRUM Co. Ltd. (Seoul, Korea) has a 13 mm thick protruded balun to match the input impedance to the feeder, and its size is 255 mm × 240 mm [16]. Recently, a coplanar waveguide (CPW)-fed square loop antenna consisting of a square loop and two circular sectors to connect the loop with central feed points was proposed to improve on the protruded balun [17]. At 210 mm × 210 mm, the antenna covered 463–1280 MHz with gain of 1.8–3.5 dBi.

In recent years, the combination of two different types of fundamental antennas were investigated extensively to increase frequency bandwidths for DTV applications. A CPW-fed wideband dipole antenna with multipole loops was proposed [18]. Five exterior loops and four interior loops were added to a printed dipole to cover the 430–1180 MHz frequency band with moderate gain of 1.8–2.8 dBi and a 241 mm × 59 mm antenna size. A miniaturized broadband antenna consisting of a microstrip-fed stubbed monopole and seven spiral lines on the ground plane was introduced for DTV reception [19]. The antenna size was very compact at 30 mm × 20 mm, but gain was very low from −19 to −12 dBi. A wideband internal planar inverted F antenna (PIFA)-loop antenna was designed on the bezel of a 49-inch TV set [20]. It consisted of a PIFA with two cutting slots and an additional shorting connector to form a loop. A PIFA mode and a loop mode of the antenna were combined to broaden the operating bandwidth. The size of the antenna was 375 mm × 17 mm with gain of 2.2–4.8 dBi. A wideband internal dipole-loop antenna with switchable and tunable frequency operation for UHD TV was proposed [21]. The dipole-loop antenna was positioned on the top edge of a 49-inch TV set. It consisted of a pair of rectangular arms (dipole mode), which were fed by the coaxial cable, and these arms were also connected to the ground plane through two shortings to generate a loop mode. A frequency reconfigurable dipole-loop antenna was achieved using PIN diodes for switchable frequency and varactor diodes for tunable frequency.

A wideband pentagonal monopole antenna was combined with a 3 × 4 high-impedance surface reflector to obtain high gain in the DTV band [22]. The unit cell of the high-impedance surface was formed by a square loop. The measured −10 dB impedance bandwidth ranged from 480 to 850 MHz with gain of 7.2–10.3 dBi. However, the size of the antenna was 460 mm × 460 mm with a height of 47 mm. A narrow-band microstrip patch antenna consisting of three series-fed patches and double-layer substrates was proposed at the center frequency of 754 MHz with a bandwidth of 25 MHz and gain of 10.5 dBi [23]. The three series-fed patches were printed on a 1.6 mm thick FR4 substrate, and an air spacing of 3.2 mm was used between the FR4 substrate and the ground plane. A CPW-fed 2 by 1 triangular patch array antenna with parasitic triangular patches was proposed for DTV reception [24]. The measured frequency band was 434–834 MHz with gain of 1.3–3.2 dBi and the antenna size of the antenna was 380 mm × 270 mm with a height of 31 mm.

Recently, an optically transparent wideband dipole and patch external antennas using metal mesh were introduced for UHD TV applications [25]. A metal mesh film with an optical transparency above 70% and a low sheet resistance of 0.04 ohm/square was used. The transparent dipole with wide arms had peak gain of 2.4 dBi, whereas the transparent patch with a capacitive feed had peak gain of 6.2 dBi. For practical receiving tests, the patch antenna was placed behind the TV set, whereas the dipole antennas were on the top and side of the TV set.

For DTV transmitting antennas, a unidirectional 1 × 4 circular bow-tie dipole array antenna with incision gaps on top of a square ground plane was proposed [26]. It covered the 450–1014 MHz frequency band with gain of 13.4–16.1 dBi in the frequency range of 470–862 MHz. A 4 × 1 antenna array with a dual-layer triangular bow-tie dipole unit was designed for DTV transmission [27]. The measured frequency bandwidth of the array antenna was 455–868 MHz with stable gain larger than 11.6 dBi over the operating frequency band.

In this paper, a miniaturized CPW-fed wideband loop antenna design for terrestrial DTV and UHD TV applications is proposed. Two different miniaturization methods (a multiple half-circular-ring-based loop structure and horizontal slits on the two circular sectors) were employed on the original wideband square loop antenna. Step-by-step design procedures for the proposed miniaturized loop antenna are provided with geometries, input reflection coefficients, and realized gain. A prototype of the proposed antenna was fabricated on a 1.6 mm thick FR4 substrate. Full-wave simulations were performed using CST Studio Suite (Dassault Systèmes Co., Vélizy-Villacoublay, France) [28].

## 2. Antenna Design

**Figure 1:**
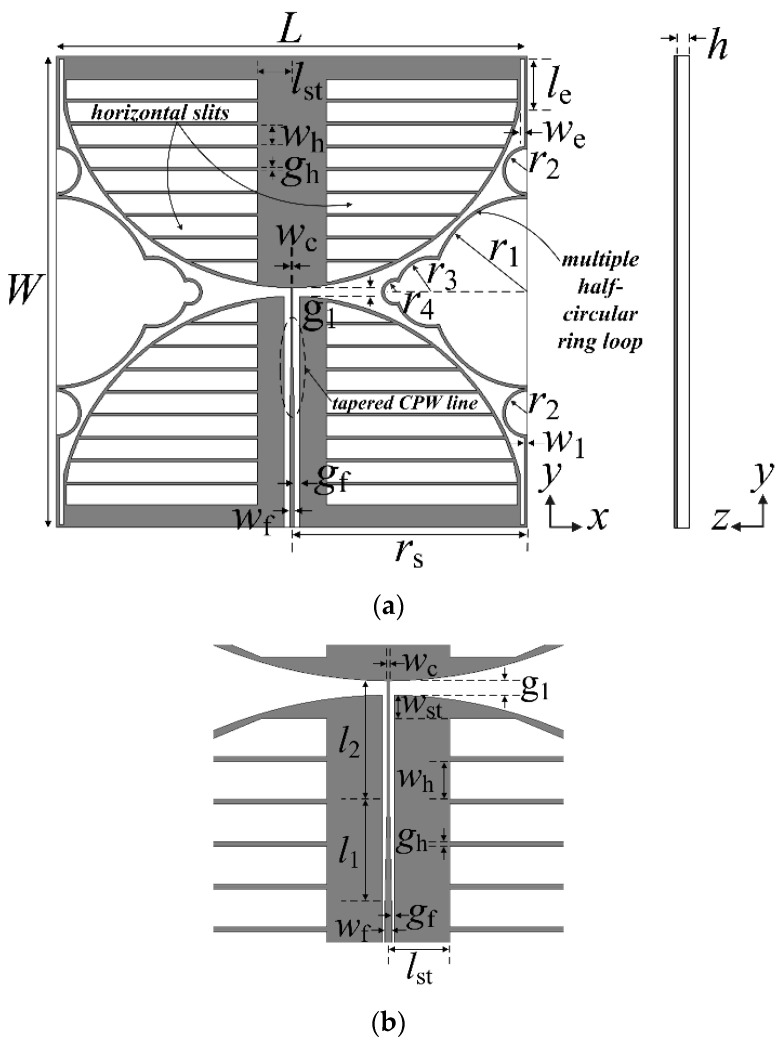
Geometry of the proposed miniaturized wideband loop antenna: (**a**) the whole view; (**b**) the tapered CPW feed line.

Figure 1 shows the geometry of the proposed miniaturized wideband loop antenna. A square loop appended with multiple half-circular rings and two circular sectors with horizontal slits are printed on one side of an FR4 substrate (*ε*_r_ = 4.4, tan *δ* = 0.025, *h* = 0.8 mm). In the lower circular sector, a CPW feed line with a tapered central signal line was inserted, and the central signal line of the CPW feed line was connected to the upper circular sector at the central feed point. The two circular sectors and the tapered CPW feed line were used to achieve the wideband characteristic. The length of the loop was increased by adding slits at the four edges where the two circular sectors and the loop meet, thereby allowing operation at a lower frequency. The length and width of the slits are denoted *l*_e_ and *w*_e_, respectively. The line width of the loop is denoted *w*_1_, and the length and width of the loop are *L* and *W*, respectively.

**Figure 2:**
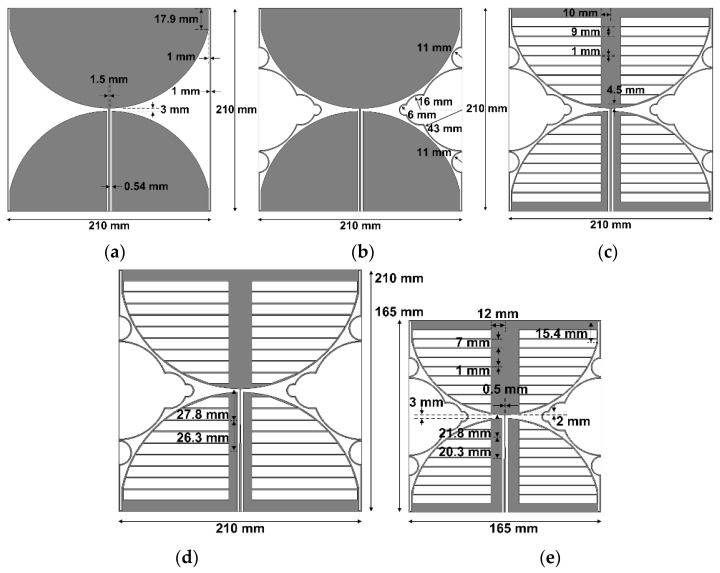
Antenna geometries considered in the design procedure for the proposed antenna: (**a**) the original CPW-fed wideband square loop antenna; (**b**) the original antenna with a multiple half-circular-ring-based loop structure; (**c**) the original antenna with the multiple half-circular-ring-based loop structure and horizontal slits; (**d**) a tapered CPW line-fed original antenna with the multiple half-circular-ring-based loop structure and horizontal slits; (**e**) the proposed miniaturized wideband loop antenna.

Spacing between the two circular sectors is *g*_1_, and the radius of the two circular sectors is half the length of the square loop. The straight side of the square loop for the original wideband loop antenna [17], shown in Figure 2a, was replaced by a multiple half- circular-ring-based loop structure. The first half-circular rings, with radius denoted as *r*_1_, were added on the straight side of the original square loop. The second half-circular rings (radius *r*_2_) are appended on both sides of the first half-circular rings. The third half-circular rings (radius *r*_3_) are inserted in the middle of the first half-circular rings. The fourth half-circular rings (radius *r*_4_) are inserted in the middle of the third half-circular rings.

The width of the center signal line at the input port of the CPW feed line is denoted *w*_f_, whereas the center signal line at the point where it meets the center feed point is *w*_c_. The spacing, *g*_f_, between the center signal line at the input port and the ground, is designed to match the 75 ohm input impedance. For impedance matching in the entire band, the width of the central signal line of the CPW feed line in the middle of the lower circular sector is linearly tapered from *w*_f_ to *w*_c_, and the length of this part is denoted *l*_1_, whereas *l*_2_ is the distance between the end of the tapered center signal line and the upper circular sector, with its width maintained as *w*_c_.

Next, horizontal slits are appended on the two circular sectors in order to further reduce the antenna size. They start at a point *l*_st_ away from the center of the circular sectors in the horizontal direction and have spacing of about 1 mm near the arc of the circular sectors. Their ends are treated with an oblique line to create a shape similar to a circular arc. The width of each horizontal slit is *w*_h_, and the spacing between the horizontal slits is *g*_h_.

Table 1 shows the final design parameters of the proposed miniaturized wideband loop antenna.

**Figure 3:**
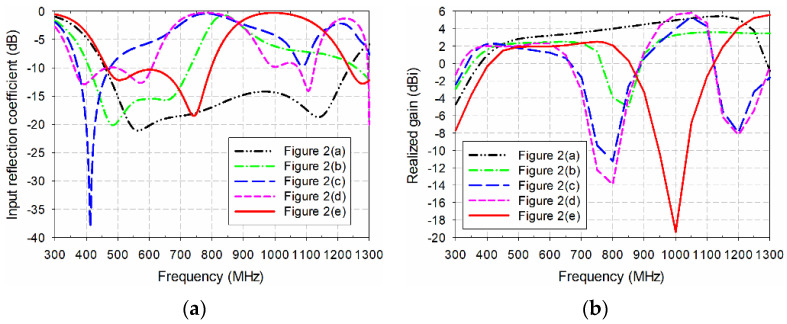
Performance comparison of the five antennas shown in Figure 2: (**a**) input reflection coefficient; (**b**) realized gain.

The design procedure for the proposed miniaturized wideband loop antenna by using the multiple half-circular-ring-based loop structure and the horizontal slits on the two circular sectors is illustrated in Figure 2. First, an original CPW-fed wideband loop antenna, used as a reference antenna, was designed to cover the DTV and UHD TV bands, as shown in Figure 2a. The length of the original CPW-fed wideband square loop antenna was 210 mm, and the length of the slits added onto the four edges was 17.9 mm. Other parameters were the same as the proposed antenna. The frequency band of the simulated input reflection coefficient, for a voltage standing wave ratio (VSWR) less than two, was 455.1–1241.4 MHz (92.7%), and gain in the band was 2.3–5.5 dBi, as shown in Figure 3. Table 2 compares the frequency band and gain for the results in Figure 3.

Secondly, the multiple half-circular-ring-based loop structure was added to the original antenna, as shown in Figure 2b. The radii of the half-circular rings were *r*_1_ = 43 mm, *r*_2_ = 11 mm, *r*_3_ = 16 mm, and *r*_4_ = 6 mm. The frequency band for a VSWR less than two was reduced to 405.4–732.5 MHz (57.5%) with shifts toward the lower frequency for the lower upper frequency limits; the gain in the band was also reduced to 1.7–2.5 dBi.

Horizontal slits were added to the antenna in Figure 2b, as shown in Figure 2c. The width of each horizontal slit and the spacing between the horizontal slits were *w*_h_ = 9 mm and *g*_h_ = 1 mm, respectively. The frequency band for a VSWR less than two was further decreased to 370.6–479.9 MHz (25.7%), with gain at 1.7–2.3 dBi.

The tapered CPW feed line was applied to the antenna in Figure 2c in order to increase impedance bandwidth. The parameters of the tapered CPW feed line were *l*_1_ = 26.3 mm, *l*_2_ = 27.8 mm, and *w*_h_ = 0.5 mm. The frequency band for a VSWR less than two was increased to 358.2–611.0 MHz (52.2%), and gain in the band was 1.7–2.3 dBi.

Finally, the proposed miniaturized wideband loop antenna was designed by reducing the geometrical parameters of the antenna in Figure 2d, which are presented in Table 1. The length of the proposed antenna was decreased to 165 mm in order to move the frequency band of the antenna in Figure 2d toward the DTV and UHD TV bands. The reduction in length of the proposed antenna compared to the original antenna was about 21.43%. Other parameters were also carefully adjusted through simulations to cover the DTV and UHD TV bands. The frequency band for a VSWR less than two increased to 460.6–799.6 MHz (53.8%), and gain in the band was 1.6–2.5 dBi.

**Figure 4:**
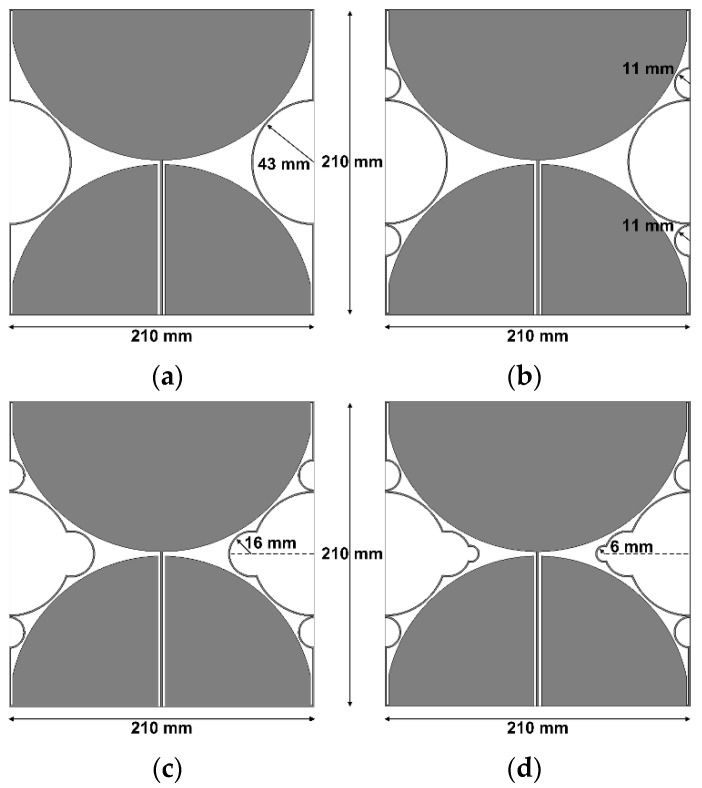
Detailed design process for the multiple half-circular-ring-based loop structure: (**a**) the original antenna with the first half-circular rings; (**b**) the original antenna with the first and second half-circular rings; (**c**) the original antenna with the first, second, and third half-circular rings; (**d**) the original antenna with the first, second, third, and fourth half-circular rings.

**Figure 5:**
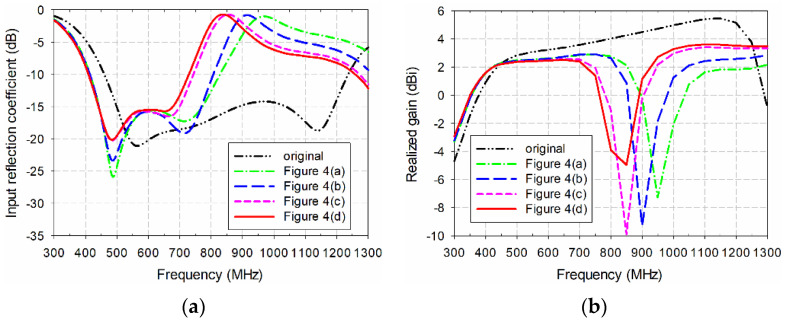
Performance comparison of the four antennas shown in Figure 4 with the original loop antenna: (**a**) the input reflection coefficients, (**b**) the realized gain.

Figure 4 analyzes a more detailed design process for the multiple half-circular-ring-based loop structure, with the corresponding performance shown in Figure 5. Table 3 compares the frequency band and gain for the results in Figure 5. When the first half-circular rings were added to the original antenna, as shown in Figure 4a, the frequency band for a VSWR less than two was reduced to 410.6–833.0 MHz (67.9%), and gain in the band was 1.8–2.9 dBi. For the original antenna with the first and second half-circular rings shown in Figure 4b, the frequency band for a VSWR less than two decreased slightly to 410.0–810.4 MHz (65.6%), but gain remained unchanged in the band. When the first, second, and third rings were appended to the original antenna, as shown in Figure 4c, the frequency band for a VSWR less than two was reduced to 406.2–746.8 MHz (59.1%), and gain was slightly reduced to 1.7–2.6 dBi. Finally, when all four types of rings were added to the original antenna, the frequency band for a VSWR less than two was reduced to 405.4–732.5 MHz (57.5%), and gain in the band was 1.7–2.5 dBi, as mentioned previously.

**Figure 6:**
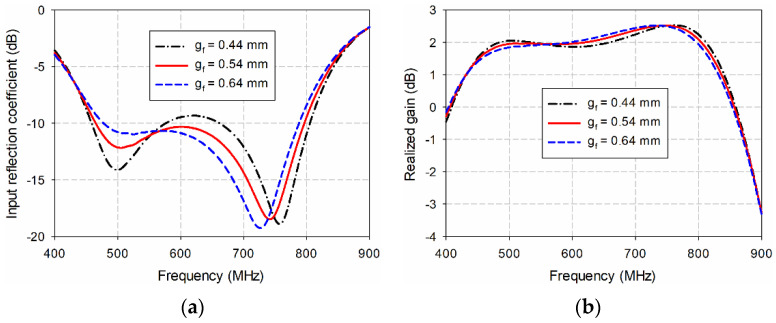
Effects of varying *g*_f_ on the performance of the proposed antenna: (**a**) the input reflection coefficients; (**b**) the realized gain.

The most sensitive geometric parameters for the performance of the proposed antenna are the spacing between the center signal line at the input port and the ground (*g*_f_), the width of the center signal line at the point where it meets the center feed point (*w*_c_), and the spacing between the two circular sectors (*g*_1_). First, the effects of varying *g*_f_ on the input reflection coefficient and gain characteristics of the proposed antenna were simulated, as shown in Figure 6. As *g*_f_ decreased from 0.64 mm to 0.44 mm, the frequency band for a VSWR less than two increased, but impedance matching in the middle band deteriorated. For example, when *g*_f_ = 0.64 mm, the frequency band was 469.9–791.4 MHz (51.0%), and gain in the band was 1.7–2.5 dBi. As *g*_f_ decreased to 0.54 mm, the frequency band increased to 460.6–799.6 MHz (53.8%), and gain in the band was 1.6–2.5 dBi. However, when *g*_f_ further decreased to 0.44 mm, the frequency band increased to 455.4–808.3 MHz (55.9%), but impedance match deteriorated in the frequency range of 594.5–643.8 MHz with a gain reduction. Therefore, *g*_f_ = 0.54 mm was chosen for the final design parameters.

**Figure 7:**
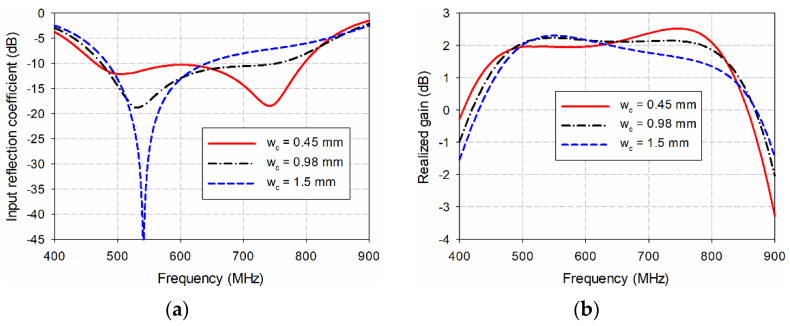
Effects of varying *w*_c_ on the performance of the proposed antenna: (**a**) the input reflection coefficients, (**b**) the realized gain.

Figure 7 shows the effects of varying *w*_c_ on the input reflection coefficient and gain characteristics of the proposed. As *w*_c_ increased from 0.45 mm to 1.5 mm, the tapered CPW feed line became the straight line and the frequency band for a VSWR less than two decreased. For instance, when *w*_c_ = 0.45 mm, the frequency band was 460.6–799.6 MHz (53.8%), and gain in the band was 1.6–2.5 dBi. As *w*_c_ increased to 0.98 mm, the frequency band decreased to 471.0–768.3 MHz (48.0%), and gain in the band was 1.7–2.2 dBi. When *w*_c_ further increased to 1.5 mm, the frequency band decreased to 481.0–651.5 MHz (30.1%), and gain in the band was 1.7–2.3 dBi.

**Figure 8:**
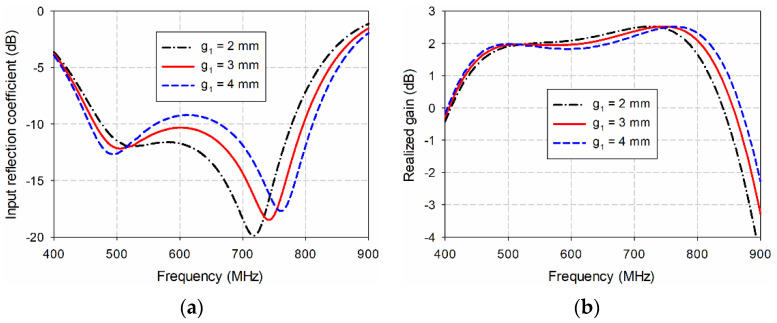
Effects of varying *g*_1_ on the performance of the proposed antenna: (**a**) the input reflection coefficients; (**b**) the realized gain.

Finally, the effects of varying *g*_1_ on the input reflection coefficient and gain characteristics of the proposed antenna were simulated, as shown in Figure 8. As *g*_1_ increased from 2 mm to 4 mm, the frequency band for a VSWR less than two increased, but impedance matching in the middle band deteriorated. For example, when *g*_1_ = 2 mm, the frequency band was 470.5–781.3 MHz (49.7%), and gain in the band was 1.7–2.5 dBi. As *g*_1_ increased to 3 mm, the frequency band increased to 460.6–799.6 MHz (53.8%), and gain in the band was 1.6–2.5 dBi. However, when *g*_1_ further increased to 4 mm, the frequency band increased to 453.1–814.3 MHz (57.0%), but impedance match deteriorated in the frequency range of 578.9–646.2 MHz with a gain reduction. Therefore, *g*_1_ = 3 mm was chosen for the final design parameters.

**Figure 9:**
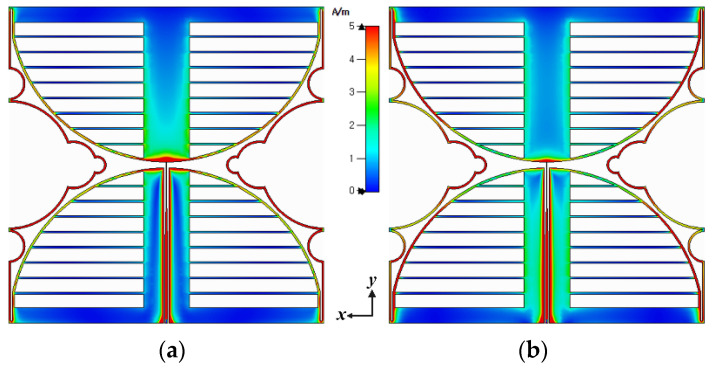
Surface current distributions of the proposed antenna at (**a**) 470 MHz, (**b**) 700 MHz.

Surface current distributions of the proposed antenna at 470 MHz and 700 MHz are shown in Figure 9. Current distributions at 470 MHz are approximately one wavelength with two maximum currents on both sides, whereas at 700 MHz, distributions are around one and a half wavelengths.

## 3. Experimental Results

**Figure 10:**
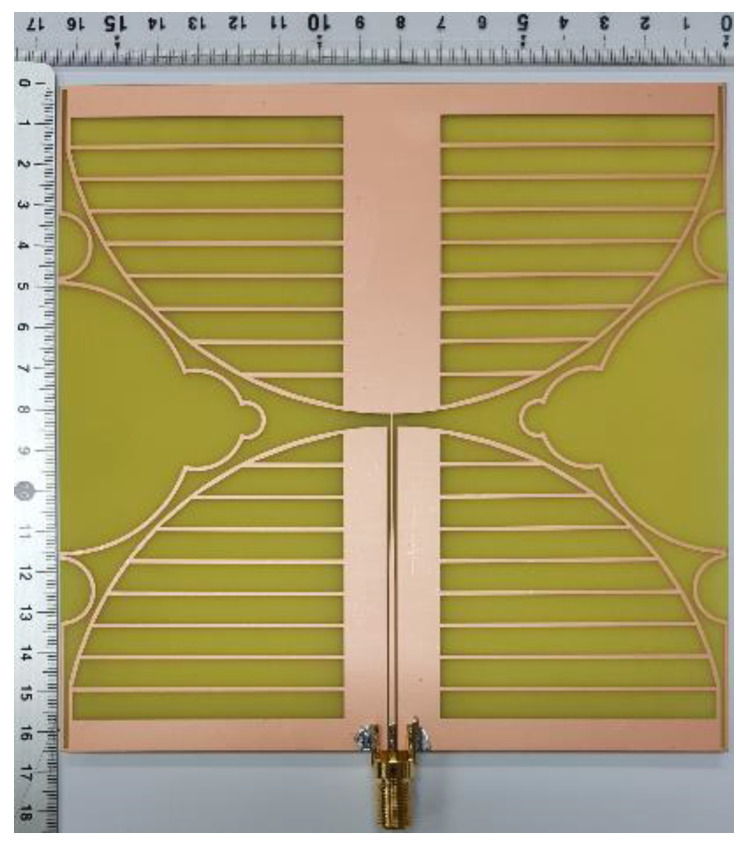
The proposed antenna fabricated on an FR4 substrate.

To validate the performance of the proposed miniaturized wideband loop antenna, it was fabricated on an FR4 substrate (*ε*_r_ = 4.4, *h* = 0.8 mm, tan *δ* = 0.025) as shown in Figure 10.

**Figure 11:**
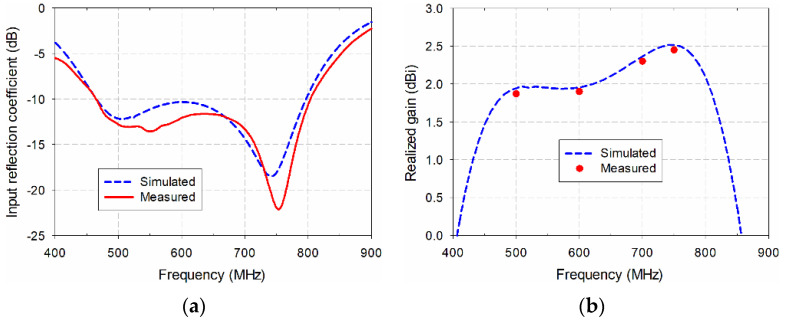
Performance comparison of the fabricated antenna: (**a**) input reflection coefficients; (**b**) realized gain.

Figure 11 compares the simulated and measured performance of the proposed antenna. An Agilent N5230A network analyzer (Santa Rosa, CA, USA) was used to measure the input reflection coefficient and the realized gain characteristics. The simulated and measured frequency bands of the proposed antenna for a VSWR less than two were 460.6–799.6 MHz (53.8%) and 460.7–806.2 MHz (54.5%), respectively. The frequency band of the measured input reflection coefficient slightly increased, compared to the simulation. The simulated gain was 1.9–2.5 dBi in the 500 MHz to 750 MHz frequency range, whereas the measured gain in the band was slightly lower than simulated gain due to errors in fabrication and measurement.

**Figure 12:**
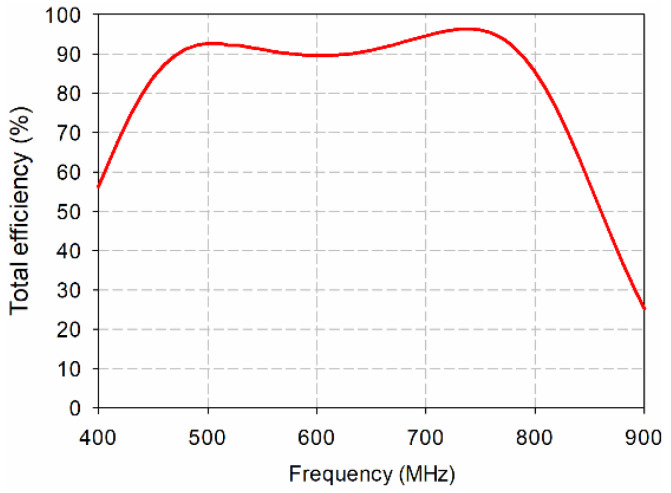
Simulated total efficiency of the proposed antenna.

**Figure 13:**
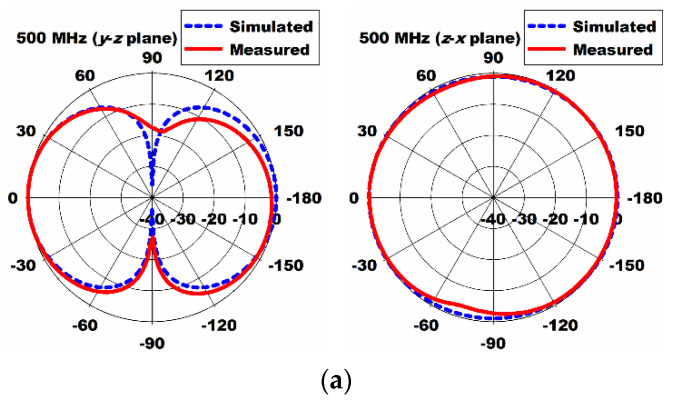

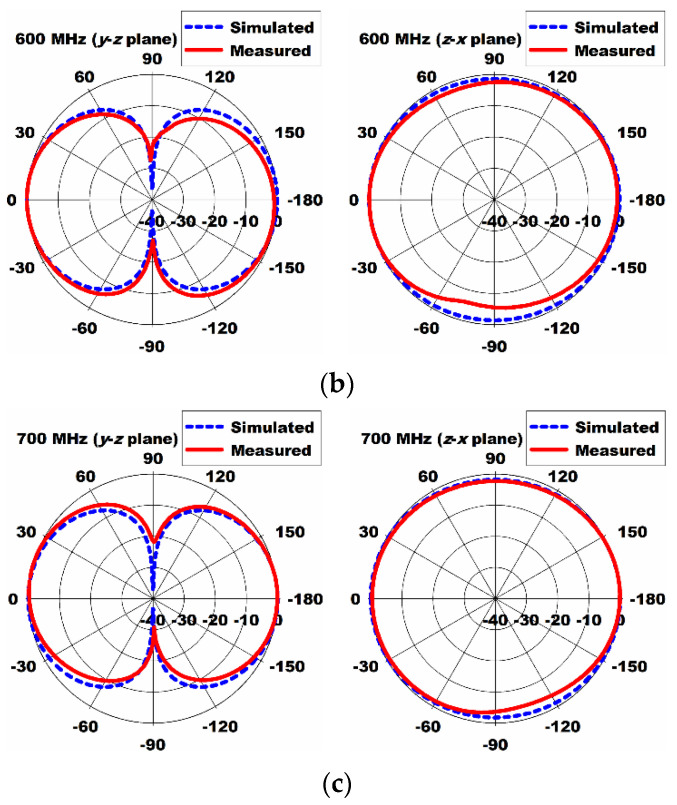
Measured radiation patterns of the fabricated antenna in the *y*–*z* and *z*–*x* planes at (**a**) 500 MHz; (**b**) 600 MHz; (**c**) 700 MHz.

**Figure 14:**
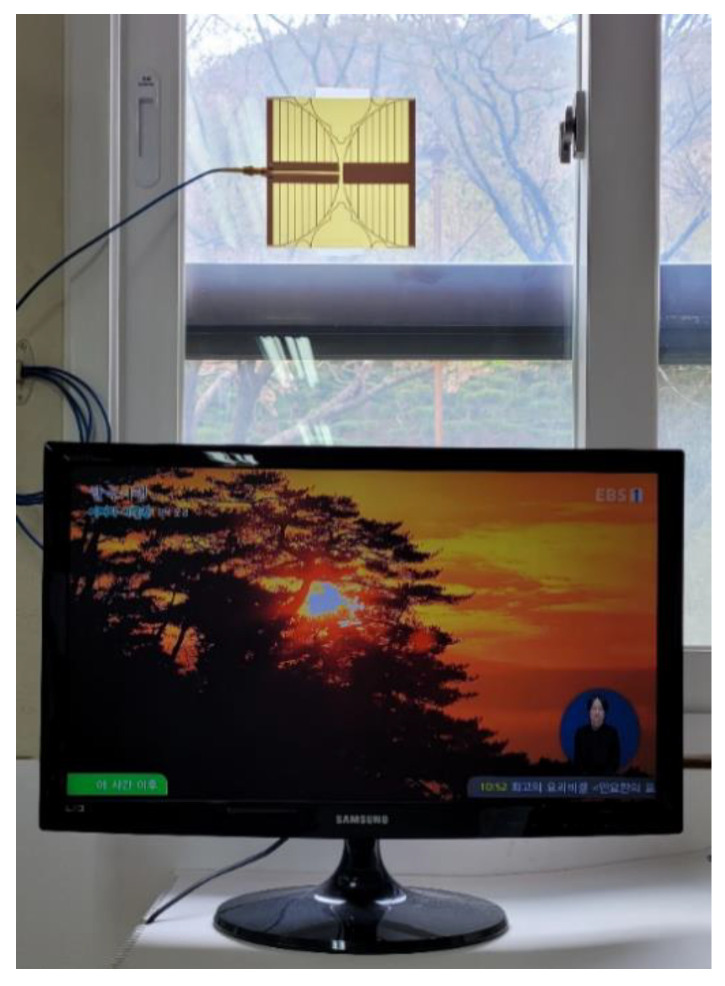
Fabricated antenna test for DTV reception inside a building when connected to a TV.

Simulated total efficiency of the proposed antenna was shown in Figure 12. It ranged from 87.2% to 96.4% in the band. The loss of the proposed antenna might be caused by the dielectric loss of FR4 substrate. The measured radiation patterns of the proposed antenna in the *y*–*z* and *z*–*x* planes at 500 MHz, 600 MHz, and 700 MHz are plotted in Figure 13. The measured radiation patterns agreed quite well with the simulated results. In order to validate DTV reception performance, the proposed antenna was tested when it was placed on a window of the office, as shown in Figure 14. All the TV channels in the DTV band were well received.

Table 4 compares the size, impedance bandwidth, and gain of the proposed antenna with those of antennas in the literature, along with the antenna type.

## 4. Conclusions

The design of a miniaturized CPW-fed wideband loop antenna for terrestrial DTV and UHD TV applications was proposed. A multiple half-circular-ring-based loop structure and horizontal slits on the two circular sectors were employed on a wideband square loop antenna in order to reduce the length.

We confirmed that the prototype antenna fabricated on an FR4 substrate at 0.8 mm thick covers the DTV and UHD TV bands with frequencies of 460.7–806.2 MHz for a VSWR less than two. The measured gain was slightly lower than the simulated results. The proposed miniaturized wideband loop antenna had a 21.43% reduction in length, compared to the original loop antenna. When the proposed antenna was tested in situ on a window of the office in order to validate DTV reception performance, all the TV channels in the DTV band were well received.

If the proposed antenna is manufactured on a flexible film substrate, it is expected that the transparency can be improved, and it can be used as a window-mounted indoor antenna. In addition, the proposed miniaturization method might be applied to design compact sensors based on antenna structures such as sensor antennas.

## Figures and Tables

**Table 1:** Parameters of the proposed miniaturized wideband loop antenna.

Parameter	Value (mm)	Parameter	Value (mm)	Parameter	Value (mm)
*L*	165	*l*_st_	12	*r*_3_	12.6
*W*	165	*w*_st_	2	*r*_4_	4.7
*w*_f_	1.5	*w*_1_	1	*w*_h_	7
*g*_f_	0.54	*l*_e_	15.4	*g*_h_	1
*w*_c_	0.5	*w*_e_	1	*l*_1_	20.3
*g*_1_	3	*r*_1_	33.8	*l*_2_	21.8
*r*_s_	82.5	*r*_2_	8.6	*h*	0.8

**Table 2:** Frequency band and gain comparison for the results in Figure 3.

Antenna	Frequency Band (MHz) for VSWR < 2	Gain (dBi)
Figure 2a	455.1–1241.4 MHz (92.7%)	2.3–5.5
Figure 2b	405.4–732.5 MHz (57.5%)	1.7–2.5
Figure 2c	370.6–479.9 MHz (25.7%)	1.7–2.3
Figure 2d	358.2–611.0 MHz (52.2%)	1.7–2.3
Figure 2e	460.6–799.6 MHz (53.8%)	1.6–2.5

**Table 3:** Frequency band and gain comparison for the results in Figure 5.

Antenna	Frequency Band (MHz) for VSWR < 2	Gain (dBi)
Original	455.1–1241.4 MHz (92.7%)	2.3–5.5
Figure 4a	410.6–833.0 MHz (67.9%)	1.8–2.9
Figure 4b	410.0–810.4 MHz (65.6%)	1.8–2.9
Figure 4c	406.2–746.8 MHz (59.1%)	1.7–2.6
Figure 4d	405.4–732.5 MHz (57.5%)	1.7–2.5

**Table 4:** Comparison among this work and other previously reported antennas in the literature.

Reference	Size (L (mm) × W (mm))	Antenna Type	Bandwidth (MHz) for VSWR < 2	Gain (dBi)
[6]	240 × 200	Quasi-Yagi	450–848	3.5–4.6
[7]	290 × 221	LPDA	460–1270	4.7–6.2
[8]	226 × 206	Yagi-Uda	470–860	4
[8]	283 × 248	LPDA	470–860	5
[9]	356 × 303	LPDA	470–790	3–9
[10]	178 × 95	Dipole	466–846	0
[11]	200 × 20	Dipole	455–1070	−0.6–1.2
[12]	250 × 45	Dipole	452–897	2.1–3.9
[13]	250 × 135	Dipole	441–890	4.7 (peak)
[14]	213 × 40	Monopole	432–827	2.19 (peak)
[15]	180 × 50	Monopole	510–790	-
[16]	255 × 240 (*H* = 13 mm)	Loop	470–806	3–4
[17]	210 × 210	Loop	463–1280	1.8–3.5
[18]	241 × 59	Dipole + Loop	430–1180	1.8–2.8
[19]	30 × 30	Monopole + Spiral lines	470–862 (VSWR < 3)	−19–−12
[20]	375 × 17	PIFA + Loop	460–870 (VSWR < 3)	2.2–4.8
[21]	1154 × 15.5	Dipole + Loop	460–780	1.6–6.4
[22]	460 × 460 (*H* = 47 mm)	Monopole + HIS	480–850	7.2–10.3
[23]	673 × 270	Microstrip patch	742–767	10.5 (peak)
[24]	380 × 270 (*H* = 31 mm)	Patch array	434–834	1.3–3.2
[25]	110 × 40	Dipole	470–771 (VSWR < 3)	2.4 (peak)
[25]	290 × 105 (*H* = 62 mm)	Microstrip patch	454–794 (VSWR < 3)	6.2 (peak)
This work	165 × 165	Loop	461–806	1.9–2.5

## Data Availability

Not applicable.
